# The Typhoid Carrier Problem

**Published:** 1909-06-19

**Authors:** 


### THE TYPHOID CARRIER PROBLEM.

A GREAT deal of attention has been attracted in recent months to the occurrence of latent typhobacillosis in apparently healthy persons who may, months or years previously, have suffered from typhoid fever. It has been proved that in a certain proportion of such individuals the intestines, fæces, gall-bladder, and so on, may continue to harbour such bacilli still capable of virulence if introduced into the alimentary tract of a susceptible person, although they do not seem to occasion any inconvenience to their actual host. Researches into these "carrier" cases and into the methods of detecting them have been carried out especially by officers of the Army in India; but it is quite evident that the typhoid-carrier may menace the public health in temperate as well as tropical zones, for typhoid fever is everywhere endemic. The mere fact that we have, as we believe, closed so many gates against the typhoid bacillus makes it the more important to secure also any newly-discovered breaches in our defences. The treatment, therefore, of the typhoid-carrier with a view to his disencumbrance of his dangerous guests is a matter of considerable importance, and until some system which will secure this is devised the most that can be done is to prevent the dissemination of such bacilli as actually escape from him. With this end in view the Medical Officer of Health for Bristol, Dr. D. S. Davies, has printed and issued a sheet of instructions to be handed to all typhoid convalescents when discharged from hospital, in which certain recommendations as to precautions to be observed are indicated for their guidance. To drive home the necessity of carrying out these precautions the sheet is prefaced by a little elementary bacteriology, and if this has the desired result of convincing the public that the advice given is based on a real knowledge of epidemic diseases and is not one more of the medical fads of which they are so suspicious, the results may be excellent. A good point is made in the paragraph which deals with certain occupations in which the handling of materials afterwards swallowed as food is prominent, such as the confectionery, bakery, and sweetmeat trades, for example.

It is too much to hope for as yet that any very considerable percentage of typhoid convalescents will take any notice of these counsels of perfection, which enjoin, amongst other things, the washing of the hands after every act of defæcation or micturition, and periodical visits to a bacteriologist for examination of the blood, excreta, etc., for the typhoid bacillus; nor, we imagine, is Dr. Davies very sanguine of immediate success in these respects. Still, glimmerings of hygiene and its bearing upon the social and economic problems of the day are slowly penetrating the general population, and the day may be less distant than is supposed when civilised society shall be educated up to appreciate its medical officers of health and to heed their simplest as well as their most elaborate warnings. On the other hand, it must not be lost sight of that the vast majority of typhoid outbreaks are definitely traceable to pollution of water, milk, and other substances derived from sources with which typhoid "carriers" have no connection. That is to say, the "carrier" factor in the promotion of epidemics in Britain must be very slight compared with that of neglect of the ordinary principles of sanitation.

